# An experimental hut evaluation of Olyset Plus, a long-lasting insecticidal net treated with a mixture of permethrin and piperonyl butoxide, against *Anopheles fluviatilis* in Odisha State, India

**DOI:** 10.1186/s12936-016-1424-1

**Published:** 2016-07-21

**Authors:** Kasinathan Gunasekaran, Sudhansu Sekhar Sahu, Tharmalingam Vijayakumar, Swaminathan Subramanian, Rajpal Singh Yadav, Olivier Pigeon, Purushothaman Jambulingam

**Affiliations:** Vector Control Research Centre (ICMR), Medical Complex, Indira Nagar, Puducherry, 605 006 India; World Health Organization Pesticide Evaluation Scheme, Department of Control of Neglected Tropical Diseases, WHO, Geneva, Switzerland; Agriculture and Natural Environment Department (D3), Walloon Agricultural Research Centre (CRA-W), Gembloux, Belgium

**Keywords:** *Anopheles fluviatilis*, Experimental-hut, Olyset Plus, Olyset Net, Odisha, India

## Abstract

**Background:**

Fast development of pyrethroid resistance in malaria vectors prompted the development of new vector control tools including combination of insecticides with different modes of action as part of resistance management strategies. Olyset Plus® is a new long-lasting insecticidal net, in which, permethrin and a synergist, piperonyl butoxide (PBO), are incorporated into filaments. Mixture nets such as this may have application against resistant mosquitoes, particularly those whose resistance is based on oxidative metabolism. There may also be enhanced activity against susceptible mosquitoes since mixed function oxidases are involved in a many metabolic activities including activation to form bioactive compounds.

**Methods:**

Bio-efficacy of Olyset Plus was evaluated against susceptible malaria vector, *Anopheles fluviatilis* in experimental huts. Deterrence, blood feeding inhibition, induced exophily and killing effect were measured to assess the bio-efficacy. The results were compared with Olyset Net®, a polyethylene permethrin-incorporated LLIN and a conventionally treated polyester net (with permethrin) washed to just before exhaustion.

**Results:**

Results showed significant reduction in entry (treatment: 0.4–0.8; control: 4.2 per trap-night) and increase in exit (56.3–82.9 % and 44.2 %) rates of *Anopheles fluviatilis* in the treatment arms compared to control (P < 0.05). While blood feeding rates declined in treatment arms (18.8–30.6 %), it increased in control (77.6 %) (P < 0.05). This was further evident from the blood-feeding inhibition rates in treatment arms (60.6–90.6 %). Total mortality was significantly higher in all treatment arms (96.3–100 %) compared to control arm (2 %) (P < 0.05). Chemical analysis for active ingredient (AI) showed retention of 75 and 88 % in Olyset plus and Olyset net respectively after 20 washes. Performance of Olyset Plus washed 20 times was equal to the CTN and Olyset Net against the susceptible malaria vector *An. fluviatilis*, fulfilling the WHO efficacy criteria of Phase II evaluation for LLIN. However, the benefit of incorporating PBO and permethrin together in a long-lasting treatment could not be demonstrated in the current study as the target vector species was fully susceptible to pyrethroids.

**Conclusion:**

Olyset Plus, with its intrinsic bio-efficacy could be an effective vector control tool to prevent transmission of malaria by susceptible vectors like *An. fluviatilis*. However, the results of the current study need to be further supported by testing the net at village level (Phase III) for community acceptability. Before taking the net to village level, it needs to be verified whether the net is better than pyrethroid nets in terms of bio-efficacy against resistant *An. culicifacies*, another malaria vector that has developed resistance to synthetic pyrethroids in India.

## Background

*Plasmodium falciparum* is the predominant human malaria parasite in India and of the total cases reported in 2015, its proportion was 67.2 % (n = 1.12 million), followed by *Plasmodium vivax* [1]. Effective and large scale implementation of conventional tools [indoor residual spraying, insecticide-treated nets (ITNs)/long-lasting insecticidal nets (LLINs), larvicides] have distinctly brought down the malaria cases from 2.08 million to 1.31 million during 2001–2011 [2]. One of the major strategies being pursued for malaria control by the National Vector Borne Disease Control Programme (NVBDCP) is the distribution of LLINs in endemic areas since 2009 [1] and so far around 21 million nets have been distributed in the country [3].

LLIN is the most technologically advanced form of insecticide-treated net currently used for malaria control [4, 5]. The LLINs, which retain insecticidal efficacy without re-treatment for 3–5 years, represent an important innovation that is being scaled up globally for malaria prevention [6–8]. These nets are made up of synthetic fibers (polyester and polyethylene) that have been compounded with an insecticide. The net kills or repels mosquitoes and it provides a physical barrier to them. Studies have demonstrated that presence of a LLIN also dejects mosquitoes from remaining in the surroundings [9]. Among the LLINs undergone trials so far, DuraNet®, Interceptor®, MAGNet™, Olyset Net®, PermaNet® 2.0, Royal Sentry® and Yorkool™ received full recommendation (a full recommendation implies that the net has undergone long-term testing under operational conditions) of World Health Organization Pesticide Evaluation Scheme (WHOPES) while DawaPlus® 2.0, LifeNet®, Olyset Plus® and PermaNet® 3.0 have been awarded with interim recommendation (an interim recommendation is granted after satisfactory completion of laboratory and small-scale field-testing of the given net) [10–12]. Two brands of LLINs viz., Olyset Net and PermaNet are already in use in some countries, including India.

Efficacy of LLINs so far relies exclusively on a single class of insecticide, synthetic pyrethroids, to which there are many reports of resistance in vector populations adopting various mechanisms [13–15]. In a multi-centre study in Western and Central Africa, field efficacy of a deltamethrin + piperonyl butoxide treated mosaic net (PermaNet® 3.0) was tested in experimental huts against pyrethroid resistant malaria vectors, *Anopheles gambiae* and *Anopheles arabiensis* and compared with PermaNet 2.0., a deltamethrin-coated LLIN. PermaNet 3.0 caused higher efficacy against the resistant malaria vectors than PermaNet® 2.0. However, in areas of strong resistance like the Vallée du Kou (Southern Burkina Faso), a large number of exposed mosquitoes survived after the exposure to both LLINs [16]. In another study in Benin, blood feeding of pyrethroid resistant *An. gambiae* was not inhibited by insecticide-treated nets, whereas inhibition was 96 % among susceptible mosquitoes. Also, the mortality rate of *An. gambiae* in resistant area was only 30 % against 98 % mortality in susceptible area [17]. Further, the household trials in northern and southern Benin showed insecticide treated nets provided little or no protection against pyrethroid resistant *An. gambiae* [18]. Due to fast development of pyrethroid resistance in malaria vectors worldwide, industries started manufacturing new vector control tools including insecticide mixtures containing at least two active ingredients with different modes of action as part of resistance management. Development of a net incorporating a pyrethroid with a synergist is promising against pyrethroid resistant malaria vectors. Synergists are chemicals that lack pesticidal effects of their own but enhance the pesticidal properties of other chemicals. One such newer vector control tool is Olyset Plus, a durable LLIN made of mono-filament polyethylene yarn, containing 2 % (w/w) technical permethrin (40:60 *cis*:*trans* isomer ratio) as active ingredient (AI), corresponding to 20 g AI/kg (about 800 mg AI/m^2^), and 1 % (w/w) piperonyl butoxide (PBO), as synergist, corresponding to 10 g PBO/kg (about 400 mg PBO/m^2^). Permethrin and the synergist are incorporated into filaments and the active ingredients slowly diffuses over the lifetime of the net to maintain a constant surface concentration. Mixture nets such as this may have application against resistant mosquitoes, particularly those whose resistance is based on oxidative metabolism [9]. There may also be enhanced activity against susceptible mosquitoes since mixed function oxidases are involved in many metabolic activities including activation of many substances to form their bio-active compounds [9]. Olyset Plus was made available by the WHOPES for Phase II evaluation (in Phase II, efficacy of washed and unwashed LLINs is evaluated in experimental huts against wild, free flying anopheline mosquitoes) in India. The current paper presents the results of the evaluation of efficacy of Olyset Plus LLIN carried out in experimental huts during 2011–2013 against a wild, free flying susceptible population of *Anopheles fluviatilis* sensu lato, in terms of mortality, deterrence, blood-feeding inhibition and induced exophily, in Odisha state, East-Central India following the WHO guidelines [19].

## Methods

### Study area

Kandhaguda village of Malkangiri District in Odisha State (East-Central India) was the field site. The terrain of the village is hilly and forested with a stream crisscrossing (altitude 150–200 m MSL). Climate is characterized by summer (March–June), rainy (July–October) and cold seasons (November–February). The village has been endemic for *P. falciparum* malaria with two transmission peaks, July–August and October–November; *An. fluviatilis* is the major malaria vector [5]. The village is under Pandripani Community Health Centre that recorded an annual parasite incidence (API) of 13.2–33.8 per 1000 population during 2013–2015. Yearly, two rounds of indoor residual spraying with DDT have been carried out in the district (Malkangiri); in addition, LLINs (PermaNet 2.0) were distributed during 2012–2013. The six experimental huts constructed in the village were used for the Phase II evaluation of Olyset Plus.

### Experimental hut

**Fig. 1:**
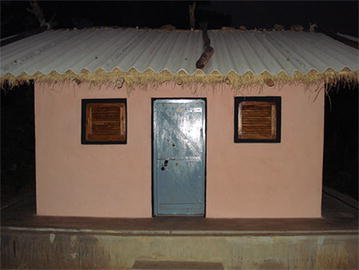
*Front view* of the experimental hut used for the Phase II evaluation

**Fig. 2:**
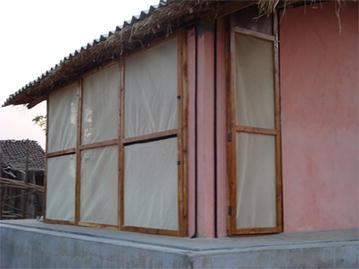
*Rear view* of the experimental hut showing verandah (exit) trap

The experimental hut is specially designed for recording the entering and exiting behaviour of mosquitoes and for measuring response to insecticides/treated nets including mortality. The hut consists of a single room with four windows; size of each window was 0.45 × 0.45 m, grilled with wooden planks fixed horizontally in tilted position one above the other leaving a gap of 1 cm between two planks through which mosquitoes could enter into the hut but could not exit. There are two windows on the front door side and one on each of the sides and a screened (using nylon mesh) verandah at the backside. The dimensions of the huts resemble to those of the village huts (length 3 m, width 3 m and height 2.5 m) having brick walls with cement plastering and thatched roof, above which there are tin-sheeted roofing for protecting the thatched roof. There is no space between the thatched ceiling and tin-roofs. The huts are constructed one foot above the ground level on a platform made up of brick and cement. The platform has a water-filled moat (6′ depth × 6′ breadth) all around to deter entry of scavenging ants. The moat is made at two feet away from the hut walls, except on the back side of the hut where it is at 1.5 ft away from the base of the verandah trap. At the centre of the hut, the roof is at a height of 2.5 m and near the wall the height is 2 m; this difference in height is to maintain a slope of the roof. The eave on the backside (facing towards east) has a gap of 1–2 cm and through this gap mosquitoes could exit, but those mosquitoes will be collected in the verandah trap. There is one wooden door of 0.75 m × 1.5 m facing towards west (Figs. 1, 2).

### Experiment arms

The test nets (size 220 cm long, 170 cm wide, 150 cm high) were received from the WHOPES. The evaluation was single blinded one as the nets were coded by a person who did not involve directly in field evaluation and the codes were not communicated to the field staff. Further, washing of nets and bioassay before and after washing were done at Vector Control Research Centre, Puducherry thereby ensuring the field staff who conducted the evaluation in experimental huts were not knowing the details of the nets. The evaluation included six comparison arms viz., unwashed Olyset Plus, Olyset Plus washed 20 times, unwashed Olyset Net, Olyset Net washed 20 times (positive control), polyester net conventionally treated with permethrin at 500 mg/m^2^ washed to just before exhaustion (reference net) and polyester untreated net (negative control). The nets were coded indicating the six arms and the six replicate nets and the two additional nets in each arm. Six replicate nets were used per each arm and each net was tested one night per week. The two additional nets were used for cone bioassay and chemical analysis prior to any wash and after 20 washes or washed to just before exhaustion.

### Washing, cone-bioassays and chemical analysis

Olyset Plus and Olyset Net were washed 20 times and the conventionally treated nets (CTN) were washed to just before exhaustion (seven nets in each Arm) [19]. The point of exhaustion was determined by washing the net and performing cone bioassay after each wash. The last wash for which the net still caused >80 % mortality or >95 % KD was considered to be the number of washes required before exhaustion. Considering a regeneration time of 2 days for Olyset Plus and 7 days for Olyset Net, the wash interval was kept respectively, as 2 and 7 days.

Bioassays were performed on the nets using the WHO-prescribed cones and the laboratory reared, blood-fed, susceptible *Anopheles stephensi* before any wash (to confirm the efficacy of the insecticide treatment of the nets) and after 20 washes (to assess the wash resistance of the treated nets) or washes until just before exhaustion at the VCRC laboratory, Puducherry. For bioassays, the target vector species, *An. fluviatilis,* could not be used as rearing facility of this species was not available at the VCRC laboratory, where net washing was done. On each net, 5 × 2 cone tests were performed (one cone on each section of the net: roof and 4 sides, repeated 2nd time) exposing five mosquitoes per cone test. Exposure to net lasted for 3 min after which mosquitoes were held for 24 h with access to sugar solution. Knock down was measured 60 min after exposure time and mortality after 24 h. Results were pooled for the 50 mosquitoes tested per net.

Prior to any wash, 5 pieces of 30 × 30 cm nettings were taken from one of the two additional nets of each of the six arms. Similarly, net samples were obtained after 20 washes or after washes until just before exhaustion (from the second additional net). Also, at the end of the experimental hut evaluation, one used-net from each arm was sampled. The samples were analyzed for insecticide content.

The nets, after washings and bioassays, were shifted to the field site for evaluation in experimental huts. Before hut evaluation, cone-bioassay using wild caught susceptible blood-fed *An. fluviatilis* was performed on one randomly selected net out of the six replicate nets from each arm. Six holes, two each on long sides and one each on front and hind ends were made (size of each hole was 4 cm × 4 cm) on all replicate nets of the six arms to simulate the conditions of a torn net and to put emphasis on testing whether the insecticide treatment, rather than the net, effectively prevents mosquito biting of sleepers. At the end of the hut evaluation, cone-bioassay, exposing field collected blood-fed *An. fluviatilis*, was conducted on one randomly selected net of each arm used in the huts.

### Selection of volunteers

Twelve village volunteers, two per hut (they would either be couples or two family members together, who would be provided with equal number of beds), were selected to sleep in the experimental huts under the mosquito net provided to them (two volunteers under one double size net,) from dusk to dawn with a small break for dinner. The volunteers (sleepers) were apprised that they should be available for the entire period of the hut evaluation and should maintain the same behavioural patterns (such as hut entering time, clothing, using bed materials, non-smoking, not making fire, sleeping under mosquito net, etc.) throughout, as these could constitute a significant source of variation that must be controlled for. Informed consent was obtained from all the volunteers and each one was remunerated. To involve human volunteers in the study, clearance was obtained from the Institute’s Human Ethics Committee.

### Hut acclimatization and suitability

For acclimatization, one adult volunteer slept overnight inside the experimental huts under an ordinary mosquito net for 1 month. Subsequently, hut suitability was ascertained by comparing the density of *An. fluviatilis* resting in the experiment huts with that in the village huts and by assessing recovery and scavenging rates. For recovery, known number of female anophelines were released into the huts in the evening and recaptured in the next day morning. To verify the presence of scavengers inside the huts, 10 dead *Anopheles* mosquitoes were placed on the floor (in four corners) of the huts in the evening, twice in a week. In the following morning, the places were checked for presence of dead mosquitoes and the available ones were picked up and counted.

### Evaluation in experimental huts

The nets of the six arms were evaluated in the six experimental huts. Each arm had six replicate nets and each net was tested one night per week. Thus, the six replicate nets of each arm were tested in one experimental hut in 1 week during six successive nights. Likewise, the replicate nets of the other five arms were evaluated in the other five experimental huts. Sleepers were organized in six teams, each with two persons. The teams formed in the beginning were not changed.

### Rotation of arms and volunteers

Using the Latin Square Rotation Scheme, the nets and the sleepers were rotated between the experimental huts. In practice, sleepers were rotated daily whereas arms weekly. Six replicate nets were used per arm and each net was tested one night during a week. In the morning, the nets were removed from the huts and stored in separate labelled bags. At the end of every week, mosquito nets together with bed materials and the white cloth spread on the floor of the room and verandah were removed from the huts. The huts were then cleaned and ventilated to remove any contamination from the nets previously used. The foot-mat and the beds (labelled arm-wise) were rotated by treatment with the arms since they came in close contact with the treated net.

### Mosquito collection

In the previous evening to each day of mosquito collection, prior to the volunteers stepped into the hut for sleeping, clean white cloths were spread on the floor of the hut (room and verandah), and the gutter all around the hut was filled with water.

The sleepers got into the experimental huts at 1900 h and remained inside until 0530 h; during that period they slept under the mosquito net provided to them. In the morning, resting and dead mosquitoes were collected separately from veranda, room (walls, roof, floor), and inside bed-net using a mouth aspirator and kept separately by hut and by collection place. The mosquitoes were identified morphologically and graded according to their abdominal condition (blood fed/unfed/semi-gravid + gravid). Alive mosquitoes were placed in wax coated paper cups (each 250 ml capacity) with access to sugar solution for 24 h and after which mortality was recorded. Mosquito collections were continued up to 12 weeks post-distribution of nets. Side effects perceived, if any, by the volunteers during or after sleeping under the nets in the experimental huts were recorded through interviews.

### Chemical analysis for active ingredient content in net samples

The net samples obtained prior to any wash, after 20 washes and at the end of hut evaluation were analysed for active ingredient content at the Walloon Agricultural Research Centre, CRA-W, Gembloux, Belgium. The 5 pieces of 25 cm × 25 cm (one from roof and four from four side panels) of each net sample were cut with scissors in 4 quarters according to the diagonal. One quarter was taken from each net piece and the 5 quarters were pooled in order to form a sub-sample representative of the net sample. The sub-sample was cut with scissors into small pieces of 5–10 mm^2^, homogenized and an analytical portion was weighed for determination of permethrin and/or PBO. A piece of 10 cm × 10 cm was also cut from the top piece in order to determine the density.

### Data analysis

The nets were decoded after completion of the hut trial. The data were analysed to determine the effect of the six arms in terms of deterrence (the number of mosquitoes caught in the hut with a treated net, as a percentage of the number in the hut with untreated net), induced exophily (increasing of exit rate [exit rate is the number of mosquitoes caught in the veranda trap, as a percentage of the total number caught in the hut including under bed-net and veranda] in the treated arm compared with the untreated arm), blood-feeding inhibition (the proportional reduction of blood feeding in huts with treated nets relative to huts with untreated nets), and total mortality (the number of mosquitoes found dead either at dawn [immediate mortality]or 24 h later [delayed mortality] as a percentage of the total numbers caught in the hut). The number of *An. fluviatilis* caught in each hut was tabulated by day and checked for variance and mean. Since, variance was greater than mean, the numbers caught (hut entry) were statistically analyzed using negative binomial regression. The exit, blood feeding and mortality rates were compared between the untreated (negative control) and the treated arms using logistic regression analysis (Stata software, Version 10). Since the total mortality was 100 % with the unwashed Olyset Net, conventionally treated polyester net and unwashed Olyset Plus, the logistic regression analysis was restricted to untreated polyester net, Olyset Net washed 20 times and Olyset Plus washed 20 times and the results were compared among these arms.

## Results

### Hut suitability

The resting density of *An. fluviatilis* in the experimental huts was about 2.5 times higher than that in the tribal huts. The recovery rate of *An. fluviatilis* was >80 % (84–91.7 %) on all the five occasions tested in the six huts. The scavenging rate was almost nil in most of the times tested, except on a few occasions in the beginning, when the scavenging rate was around 3 %. Although, the huts were made ant-proof by filling the gutter around the hut with water, house crickets, *Gryllodes sigillatus* were found scavenging on dead mosquitoes; this problem was overcome by cleaning the rooms, verandah and surroundings of the huts every day.

### Species composition

From the 72 nights of collections per experimental hut, i.e. six nights in a week for 12 weeks, the total mosquito catch was 965. About 53 % of them were *An. fluviatilis*. *Anophelesculicifacies,* the secondary malaria vector, formed only 1.9 %; 14.9 % was other anophelines and 29.8 % culicines. Since, *An. culicifacies* was collected in very low numbers; further analysis was done only for *An. fluviatilis*. Results of the entomological collections and statistical analysis are summarized, respectively in Tables 1 and 2.

**Table 1:** Comparison of performance of the six experimental arms against wild *Anopheles fluviatilis* in experimental huts

Arms	Untreated polyester net	Unwashed Olyset Net	Conventionally treated polyester net	Olyset Net washed 20 times	Olyset Plus washed 20 times	Unwashed Olyset Plus
Number of collections	72	72	72	72	72	72
Number of females entered/caught	303	49	41	54	36	32
Number of females caught per night	4.2^a^	0.7^b^	0.6^b^	0.8^b^	0.5^b^	0.4^b^
Deterrence in % (95 % CI)	–	83.8^a^ (79.7, 88.0)	86.5^a^ (82.6, 90.3)	82.2^a^ (77.9, 86.5)	88.1^a^ (84.5, 91.8)	89.4^a^ (86.0, 92.9)
Exit rate in % (95 % CI)	44.2^a^ (38.6, 49.8)	69.4^b^ (56.5, 82.3)	82.9^b^ (71.4, 94.4)	70.4^b^ (58.2, 82.5)	77.8^b^ (64.2, 91.4)	56.3^ab^ (39.1, 73.4)
Blood feeding in % (95 % CI)	77.6^a^ (72.9, 82.3)	26.5^b^ (14.2, 38.9)	7.3^b^ (1.5, 19.9)*	29.6^b^ (17.5, 41.8)	30.6^b^ (15.5, 45.6)	18.8^b^ (5.2, 32.3)
Blood feeding inhibition in % (95 % CI)	–	65.9^a^ (45.3, 78.6)	90.6^a^ (71.9, 96.8)	61.9^a^ (42.1, 74.8)	60.6^a^ (35.3, 76.0)	75.8^a^ (50.1, 88.3)
Total mortality in % (95 % CI)	2.0^a^ (0.4, 3.5)	100.0^b^ (100, 100)	100.0^b^ (100, 100)	96.3^b^ (87.2, 99.6)*	97.2^b^ (85.5, 99.9)*	100.0^b^ (100, 100)

Numbers in the same row sharing a letter superscript do not differ significantly (P > 0.05)

Total mortality = Immediate mortality + Delayed mortality

Confidential intervals (CIs) for percentages are based on normal approximation to binomial distribution, except the CIs marked with asterisk that are based on exact binomial distribution

**Table 2:** Results of statistical analysis of the performance of the six experimental arms against wild *An. fluviatilis* in experimental huts

Variables	Untreated polyester net^a^	Unwashed Olyset Net	Conventionally treated polyester net	Olyset Net washed 20 times	Olyset Plus washed 20 times	Unwashed Olyset Plus
Entry (deterrence)						
IRR (95 % CI)	1.0	0.16 (0.10–0.26)	0.14 (0.08–0.22)	0.18 (0.11–0.28)	0.12 (0.07–0.20)	0.11 (0.06–0.18)
Z		−7.61	−8.08	−7.33	−8.38	−8.61
P		<0.001	<0.001	<0.001	<0.001	<0.001
Exit (induced exophily)						
Odds ratio (95 % CI)	1.0	2.86 (1. 50–5.47)	6.13 (2.63–14.25)	2.99 (1.60–5.61)	4.41 (1.95–10.00)	1.62 (0.78–3.38)
Wald statistics		10.08	17.70	11.78	12.66	1.67
P		0.001	<0.001	0.001	<0.001	0.197
Blood feeding (inhibition)						
Odds ratio (95 % CI)	1.0	0.10 (0.05–0.21)	0.02 (0.01–0.08)	0.12 (0.06–0.23)	0.13 (0.06–0.27)	0.07 (0.03–0.17)
Wald statistics		41.25	37.72	41.12	28.34	32.69
P		<0.001	<0.001	<0.001	<0.001	<0.001
Total mortality (killing)						
Odds ratio (95 % CI)	1.0	^b^	^b^	1287.0 (252.9–6550.3)	1732.5 (202.7–14810.7)	^b^
Wald statistics				74.38	46.40	
P				<0.001	<0.001	

^a^Reference category for all variables, *IRR* incidence rate ratio

^b^As the mortality was 100 % in these arms, they were excluded from the logistic regression analysis

### Entry

In the huts with the untreated control net, mosquito entry was zero on 10 occasions (out of 72 nights of collections), whereas with the five treatments, zero catch was recorded on 47, 44, 46, 51 and 54 occasions, respectively, indicating a marked deterrent effect of permethrin. While, 303 *An. fluviatilis* entered the control arm (with untreated net), the number entered the five treatment arms together was only 212. The deterrent effect of permethrin was further evidenced from a higher catch of *An. fluviatilis* under untreated net (57 of 303) compared to a very few catch under treated nets (1/49, 0/41, 0/54, 2/36 and 1/32, respectively) in spite of six holes made in all treated and untreated nets to simulate the conditions of a torn net.

The outcome of the negative binomial regression analysis (performed with the number caught as dependent variable and the six experiment arms as independent variable) revealed that the hut entry of *An. fluviatilis* was over-dispersed (non-random) (α that measures over-dispersion = 1.21 (95 % CI: 0.87–1.67), χ^2^ = 190.35, df = 5, p < 0.0001) and justified the analysis. The entry was significantly lower with all treatments compared to the control (p < 0.05). Among the five treated arms, the lowest entry was with unwashed Olyset Plus followed by Olyset Plus washed 20 times (Table 1). However, the 95 % CI for the incidence rate ratio (IRR) indicated no significant difference between the treatment arms (Table 2).

### Exit

The exophily from the control hut (natural exophily) was 44.2 % and from the huts with treated nets it ranged from 56.3 to 82.9 %. The number exited on each day in each arm during the evaluation period was subjected to logistic regression analysis by taking mosquito exit as dependent variable, the six arms as categorical covariates and the untreated net as reference category. All treatments induced significantly higher exophily than the untreated net (p < 0.05), except the unwashed Olyset Plus (p > 0.05) (Table 1). However, there was no significant difference between the five treatments, as shown by 95 % CI for the odds ratio (Table 2).

### Blood feeding

Overall, the blood feeding rate differed significantly between the six arms (Wald statistics = 125.7, p < 0.001). Though, none of the treatments inhibited blood-feeding completely, blood feeding inhibition (BFI) rates were significantly higher with all treatments compared to the control (p < 0.001) (Table 1). Among the treatments, BFI was the highest with the conventionally treated net followed by unwashed Olyset Plus; however, the 95 % CIs for the odds ratio indicated no significant difference among the five treatments (Table 2).

### Mortality

Immediate mortality rate was nil with the control net during the entire evaluation period while with the treatments it ranged from 14.6 to 40.6 %, the highest killing was with unwashed Olyset Plus. Similarly, total mortality rate was significantly higher with all treatments (96.3–100.0 %) than the untreated net (2 %) (Table 1). Results of logistic regression analysis (arms with 100 % total mortality were not included in the analysis: unwashed Olyset Net, conventionally treated polyester net, unwashed Olyset Plus) showed that the mortality was significantly higher with Olyset Net washed 20 times (OR and 95 % CI: 1287.0 and 252.9–6550.3) and Olyset Plus washed 20 times (1732.5 and 202.7–14,810.7) compared to untreated polyester net (Table 2). However, Olyset Net and Olyset Plus after washing 20 times did not differ significantly (95 % CI for the odd ratios overlap), indicating their similar killing effect.

### Residual activity of the insecticide on the nets

Prior to any wash, all treatments caused 100 % mortality of *An. stephensi* while mortality was nil with the untreated net. After 20 washes, mortality with Olyset Net and Olyset Plus was 62 % and 90 %, respectively. Prior to use in the experimental huts, mortality of *An. fluviatilis* was 100 % with all LLIN treatments and 86.0 % with the conventionally treated net; only 2.0 % mortality with the untreated net. After the hut evaluation, mortality was 100 % with all LLIN treatments and 96.0 % with the conventionally treated net; mortality was 4 % with untreated net (Table 3).

**Table 3:** Results of cone-bioassays

Sl no	Arms	Before any wash		After 20 washes		Prior to hut evaluation		After hut evaluation	
		NE	CM (%)	NE	CM (%)	NE	CM (%)	NE	CM (%)
1	Untreated polyester net	50	0	50	0	50	2	50	4
2	Unwashed Olyset Net	50	100	–	–	50	100	50	100
3	Polyester net conventionally treated, washed until just before exhaustion	50	100	50	82^a^ (4 washes)	50	86	50	96
4	Olyset Net washed 20 times	50	100	50	62	50	100	50	100
5	Olyset Plus LLIN washed 20 times	50	100	50	90	50	100	50	100
6	Unwashed Olyset Plus LLIN	50	100	–	–	50	100	50	100

*An. stephensi* was used for cone-bioassays before any wash and after washes, whereas prior to hut evaluation and after hut evaluation, *An. fluviatilis* was used for the cone-bioassays

*NE* number of mosquitoes exposed, *CM* corrected mortality

^a^Washed until just before exhaustion

### Insecticide content

Prior to washing, the permethrin content in two samples of unwashed Olyset Net (19.9 and 20.0 g/kg) and Olyset Plus (19.1 and 18.8 g/kg) complied with the target dose of 20 g/kg (±3 g/kg) and 20 g/kg (±25 %), respectively. In Olyset Net and Olyset Plus washed 20 times, the permethrin content was 17.7 and 14.1 g/kg, respectively, corresponding to an overall active ingredient retention of 88 and 75 %, respectively. The PBO content in two samples of unwashed Olyset Plus (9.0 and 8.8 g/kg) complied with the target dose of 10 g/kg (±25 %). The PBO content was 3.96 g/kg after 20 washes, corresponding to an overall PBO retention of 45 % (Table 4).

**Table 4:** Results of chemical analysis

Treatment	Before washing	After washing	AI retention (% of wash 0)	After testing
Permethrin content (g/kg)				
Unwashed Olyset Net	19.94	–	–	19.34
Olyset Net washed 20 times	20.01	17.68	88 %	17.58
Unwashed Olyset Plus LLIN	19.13	–	–	17.64
Olyset Plus LLIN washed 20 times	18.82	14.11	75 %	13.75
Polyester net conventionally treated, washed until just before exhaustion	15.33	11.36	74 %	10.42
Untreated polyester net	<1	<1	–	<1
Piperonyl butoxide content (g/kg)				
Unwashed Olyset Net	<1	<1	–	<1
Olyset Net washed 20 times	<1	<1	–	<1
Unwashed Olyset Plus LLIN	9.02	–	–	8.26
Olyset Plus LLIN washed 20 times	8.79	3.96	45 %	3.84
Polyester net conventionally treated, washed until just before exhaustion	<1	<1	–	<1
Untreated polyester net	<1	<1	–	<1

The unwashed CTN contained 509.4 mg/m^2^ (15.3 g/kg) permethrin and after washing to just before exhaustion the permethrin content was 370.3 mg/m^2^ (11.4 g/kg), corresponding to a retention rate of 74 %. After the experimental hut evaluation, the permethrin and/or PBO content in the tested Olyset Net and Olyset Plus did not decrease significantly.

## Discussion

The first LLIN to receive full recommendation of the WHOPES [20] was Olyset Net; since then, more brands of nets have been granted either interim or full recommendation. However, all the nets including Olyset Net are produced using a single class of insecticide, pyrethroids, because of their promising activity against mosquitoes and low mammalian toxicity.

In view of vectors developing resistance to pyrethroids, manufacturers produce new vector control tools/products including mixtures of insecticides comprising at least two active ingredients having dissimilar mode of action as part of resistance management strategy. Olyset Plus was the first LLIN to incorporate PBO, a synergist, into its every fibre and all surfaces, facilitating enhanced knock down and kill against pyrethroid-resistant mosquitoes. PBO has long been used to improve the performance of pyrethroid insecticides especially household aerosols [21]. Synergists are chemicals that do not possess insecticidal activity of their own but enhance the insecticidal performance of other chemicals.

Insects, in general, despite their susceptible or resistance status, contain enzymes for metabolizing xenobiotic compounds (insecticides) and converting them to a non-toxic ones that are finally removed through excretion. Cytochrome P450s are one such oxidising enzymes that detoxify pyrethroids before the preferred effect is attained. In resistant insects the activity of these enzymes can greatly be enhanced which can significantly reduce the efficacy of an insecticide. PBO is a potent inhibitor of these enzymes blocking/nullifying their action thereby inhibiting the breakdown or the metabolism of insecticides, rendering the insecticide more effective. PBO also increases the activity of pyrethroids in susceptible insects, so adding PBO to a LLIN has a benefit even in areas where there is no resistance. Many studies demonstrated the impact of PBO on pyrethroid resistance; the net incorporating permethrin and PBO showed significantly better performance in terms of insecticidal activity against multiple resistant (to permethrin) populations of *An. gambiae* than the net treated only with permethrin [9, 22].

The current experimental hut evaluation of Olyset Plus was conducted in an area where *An. fluviatilis* was the primary vector of malaria and susceptible to pyrethroids [5]. Prior to any wash, all treatments, including the two LLIN treatments (Olyset Plus and Olyset Net), caused 100 % mortality of *An. stephensi*. After 20 washes, the mortality rates induced by the LLIN treatments declined, much with Olyset Net (100–62 %) than with Olyset Plus (100–90 %). However, the insecticidal effect of the LLIN treatments regained subsequently to cause 100 % mortality of *An. fluviatilis* prior to and after the hut evaluation. The reduced mortality after washing could also be due to the difference in physiological response of the two vector species to the pyrethroid though they were phenotypically susceptible to the insecticide. With the conventionally treated net (CTN) also, after washing four times (number of washes required before exhaustion) mortality of *An. stephensi* declined from 100–82 %; however, prior to and after the hut trial, mortality of *An. fluviatilis* induced by the CTN was on higher side, 86 and 96 %, respectively.

Relatively higher efficacy of Olyset Plus (both washed and unwashed) in terms of deterrence (preventing mosquito entry) was confirmed compared to the standard Olyset Net and the untreated control. But, the same LLIN treatment, when tested against the moderate pyrethroid resistant *An. gambiae,* there was no significant reduction in the entry rates (deterrence) [9]. The unwashed Olyset Plus conferred a higher inhibition of blood feeding than Olyset Net, but after 20 washes the inhibition effect was almost equivalent to washed and unwashed Olyset Net. The induced exophily was significantly lower with unwashed Olyset Plus compared to Olyset Net as observed for *An. gambiae* in Benin during an experimental hut study [9]. But, interestingly, after 20 washes, Olyset Plus induced higher exophily than Olyset Net. Another combination LLIN, PermaNet 3.0, has also been reported to induce more exophily after 20 washes [16]. In terms of insecticidal activity (killing effect), all treatments were comparable, but caused significantly high mortality compared to the control.

The decline in permethrin content in Olyset Plus (18.8–14.1 g/kg) with 75 % retention after 20 washes indicated depletion of bio-availability of AI on the net surface by the washes. In the case of Olyset Net, the depletion, after 20 washes, was relatively lower (20.0–17.7 g/kg) as the AI retention was 88 %. In contrast, Gimnig et al. [23] reported no significant decrease of permethrin content in Olyset Net after washing as its biological activity could be restored by heat-assisted regeneration. In the current study, although there was a reduction in permethrin content of Olyset Plus after washing, it was not reflected from its performance against the susceptible vector species in the experimental hut, as it produced comparable effect. The AI retention rate of PBO in Olyset Plus was 45 % after 20 washes.

## Conclusion

The evaluation in experimental huts demonstrated that the performance of Olyset Plus washed 20 times was equal to the conventionally treated net and Olyset Net in terms of deterrence, induced exophily, blood-feeding inhibition and killing effect against the malaria vector species and thereby fulfilled the WHO efficacy criteria of Phase II evaluation for LLIN. However, the benefit of incorporating PBO and permethrin together in a long-lasting treatment could not be demonstrated in the current study as the target vector species was fully susceptible to pyrethroids. In India, among the six (*An. culicifacies*, *An. stephensi*, *An. fluviatilis*, *Anopheles minimus*, *Anopheles baimaii* and *Anopheles sundaicus*) primary vectors of malaria, *An. culicifacies*, which is a rural and peri-urban vector contributing to around 65 % of the malaria cases [24], has developed wide-spread resistance to DDT and malathion and recently to synthetic pyrethroids in many states [25–28]. Except *An. stephensi*, which has also developed wide-spread resistance to DDT and malathion, the other vector species are susceptible to all the insecticides. Olyset Plus, with its intrinsic bio-efficacy could be an effective vector control tool to prevent transmission of malaria by susceptible vectors like *An. fluviatilis* in India. With the potential benefit of incorporating a synergist [9], it could also be considered for use in areas of the pyrethroid resistant vector, *An. culicifacies*. However, before any community level testing is done, this net should be tested at hut scale against *An. culicifacies.* Further, although pyrethroid resistance is a problem with only *An. culicifacies* in India, considering the quantum of malaria it transmits, it would be essential to promote use of PBO mixed pyrethroid nets for an effective control of malaria in rural areas.

## Abbreviations

PBO: piperonyl butoxide
LLINs: long-lasting insecticidal nets
WHO: World Health Organization
ITNs: insecticide-treated nets
IRS: indoor residual spray
WHOPES: World Health Organization Pesticide Evaluation Scheme
MSL: mean sea level
API: annual parasite incidence
CTN: conventionally treated net
OR: odds ratio
CI: confidence interval
IRR: incidence rate ratio
BFI: blood feeding inhibition
